# Letter to the editor: Bias in the vaccine effectiveness estimates of one-dose post-exposure prophylaxis against mpox

**DOI:** 10.2807/1560-7917.ES.2023.28.34.2300358

**Published:** 2023-08-24

**Authors:** Catharina E van Ewijk, Susan JM Hahné

**Affiliations:** 1 Centre for Infectious Disease Control, National Institute for Public Health and Environment (RIVM), Bilthoven, the Netherlands; 2 European Programme for Intervention Epidemiology Training (EPIET), European Centre for Disease Prevention and Control (ECDC), Stockholm, Sweden

**Keywords:** sexually transmitted infections, viral infections, vaccines and immunisation, epidemiology

**To the editor: **We read with interest the article by Montero Morales et al. [1] regarding the post-exposure vaccine effectiveness of the Modified Vaccinia Ankara–Bavarian Nordic (MVA-BN) vaccine against mpox disease in contacts of mpox cases.

Vaccine effectiveness studies are important to inform policymakers how best to allocate scarce resources. The authors estimated an adjusted vaccine effectiveness (VE) of one-dose MVA-BN as post-exposure prophylaxis (PEP) of 89% (95% CI: 76–95%). We think the estimated VE is overestimated because of bias.

Mpox has a median incubation period of 7–9 days (range: 1–26 days) [2-6], which is short compared with the time it takes to generate vaccine-induced antibodies (2–4 weeks) [7]. The window of opportunity for PEP to prevent mpox in contacts is therefore very brief and for some even non-existent. This is a problem for implementing PEP and also makes it difficult to estimate VE without bias and to interpret the resulting VE.

In the article of Montero Morales et al., two key pieces of information are lacking. Firstly, it is unclear why certain contacts were not offered PEP. Secondly, the timeliness of Public Health consultation and PEP administration relative to the exposure is not described. We suspect that a considerable proportion of contacts who became a case already had mpox disease manifestations before they had the chance to be vaccinated, as Supplementary Figure 1 (Kaplan–Meier survival curves for vaccinated and unvaccinated contacts), suggests. Indeed, Table 4, which shows univariate and multivariate analysis of one-dose mpox vaccine effectiveness, suggests that most vaccinated contacts got their vaccination 7–13 (or more) days after exposure, a timeframe which includes the median incubation period. The contacts who received PEP represent a group that excludes those who had already become an mpox case. This group, therefore, has a smaller chance of disease than the unvaccinated contacts. This means that the estimated VE would be an overestimation of the true VE. The sensitivity analysis by Montero Morales et al. is inadequate to address this, since it only refers to the small proportion of contacts who made an appointment for vaccination and not to the contacts that were not offered or did not accept vaccination.

Furthermore, the analysis of Montero Morales et al. seems affected by immortal time bias, since contacts who received PEP could not have developed mpox symptoms before they received PEP. When follow-up time starts before the intervention (i.e. PEP), which seems to be the case in the article of Montero Morales et al., the person is ‘immortal’ in the period before the intervention: in this period the outcome, by definition, cannot occur. When this immortal time is misclassified or excluded, immortal time bias occurs [8]. This can be accounted for by including vaccination in the model as a time-varying exposure, i.e. time until vaccination is counted as unvaccinated person time. This would already substantially reduce the estimated VE. However, also the time it takes for the vaccine to become effective should be accounted for, which would reduce the VE even further.

Still, even if immortal time bias and the confounding described above had been properly taken into account, the question remains to whom the estimated VE applies. Because of the short incubation period of mpox and the timing of PEP administration, the estimated VE would be restricted to individuals who developed symptoms late enough to be contacted by the Public Health Service and receive PEP before that. The contacts who received PEP likely differ substantially from contacts who did not in terms of number, timing, type and intensity of exposure and therefore have a smaller chance of disease, i.e. an additional source of confounding, biasing the VE estimate upwards. The VE would only include vaccinated contacts who were in the tail end of the incubation period. This limits the generalisability of the observed VE to all contacts.

We believe that the presence of confounding and immortal time bias will have led to an overestimation of the VE in the article of Montero Morales et al. and will give a misleading impression on the benefit that PEP can offer to prevent mpox after exposure.
